# Analysis of emission of volatile organic compounds and thermal degradation in investment casting using fused deposition modeling (FDM) and three-dimensional printing (3DP) made of various thermoplastic polymers

**DOI:** 10.1007/s11356-024-35200-x

**Published:** 2024-10-09

**Authors:** Aleksander Hejna, Mariusz Marć, Paweł Szymański, Kamila Mizera, Mateusz Barczewski

**Affiliations:** 1 Polymer Processing Division, Institute of Materials Technology, Faculty of Mechanical Engineering, Poznan University of Technology, Piotrowo 3, Poznan, 61-138 Poland; 2 Faculty of Chemistry, Department of Analytical Chemistry, Gdansk University of Technology, Gabriela Narutowicza 11/12, 80-233 Gdansk, Poland; 3 Division of Foundry, Institute of Materials Technology, Faculty of Mechanical Engineering, Poznan University of Technology, Piotrowo 3, Poznan, 61-138 Poland; 4 Department of Chemical, Biological and Aerosol Hazards, Central Institute for Labour Protection – National Research Institute, Czerniakowska 16, 00 – 701 Warsaw, Poland

**Keywords:** FDM, 3DP, VOC emission, Thermal analysis, Investment casting

## Abstract

The study examined the degradation process of various types of polymers used to form models using the fused deposition modeling (FDM) and three-dimensional printing (3DP) methods and used in the investment casting method. Commercial filaments made of polylactide (PLA), acrylonitrile butadiene styrene terpolymer (ABS), high-impact polystyrene (HIPS), polyamide 12 (PA12), poly(methyl methacrylate) (PMMA), and polypropylene (PP) were used to produce gypsum molds. The assessment included organic volatile compounds (VOCs) released during mold heating and model degradation, which is characteristic of this technological process. The screening qualitative chromatographic analysis of decomposition products sampled from various points of the production hall made it possible to define potential threats related to the processing of selected polymers and the necessary preventive measures. Furthermore, passive diffusion-type samplers were used at the sampling stage of VOCs emitted to the gaseous phase to reduce nuisance and user interference. Studies have been completed to characterize the thermoplastic polymer degradation process. The coupled thermogravimetry (TGA) with analysis of gaseous products by infrared spectroscopy with Fourier transformation (FT-IR) has been used. The presented results are the first to compare and rate the use of this still-developing novel aspect of castings by the method of fused models.

**Graphical abstract:**

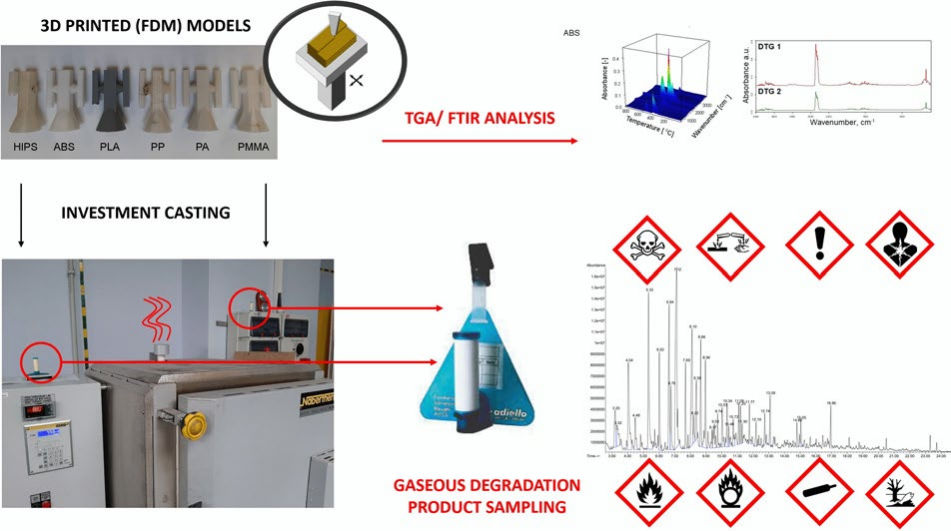

**Supplementary information:**

The online version contains supplementary material available at 10.1007/s11356-024-35200-x.

## Introduction

Investment casting (IC), although the first examples of the use of this technique can be found many centuries ago, and significant development was recorded at the beginning of the twentieth century, is still a technology that is being developed and creates opportunities to implement new innovative solutions and material and technological modifications (Jones and Yuan 2003; Prakash et al. 2021). This technology allows for obtaining high-quality metal parts with outstanding surface quality and dimensional reproduction of the models used (Kroma et al. 2020). In the most common variant, wax (Hock et al. 2003) is used as the model and pattern material. Its use enables the recovery, modification, and recycling of modeling material, which makes this technology, in a sense, sustainable (Grześkowiak et al. 2015; Czarnecka-Komorowska et al. 2020). For small production series, which low production preparation costs must characterize, porous materials are used, such as polyurethane (PUR) or expanded polystyrene (EPS) (Zhao et al. 2019). The fundamental difference between using wax and thermoplastic/thermoset polymers in investment casting is that melted wax is removed from the mold during its heating. When polymeric models are removed from the mold, they are fired, which is related to releasing thermal degradation products into the atmosphere.

The intensive development of 3D printing technology over the last 20 years has revolutionized almost all industries. The development of new materials and their forming technologies has created the possibility of rapid prototyping of products with complex shapes, allowing for the manufacturing of products with increasingly better properties and quality. 3D printing had the most significant impact on foundry technologies when replacing traditional wax models in investment castings. The use of models made of thermoplastics as well as chemical and photocurable materials, molded using technologies such as fused deposition manufacturing (FDM) (Tiwary et al. 2019; Choe et al. 2022), selective laser sintering (SLS) (Yang et al. 2009), or stereo-lithography (SL) (Li et al. 2015; Mukhtarkhanov et al. 2020), has been described in the literature. The use of 3D printing technology allows for customization of the geometry of models and, therefore, metal products without the need to produce expensive metal molds for forming wax models. In many cases, this allows for the optimization of product geometry and a significant reduction in production costs in the case of small production series.

From the point of view of maintaining good-quality castings, it is essential to completely remove the remains of lost models from the mold cavity (Yang et al. 2009). At the same time, in the case of forming thin-walled products, it is necessary to use polymeric materials with higher stiffness and mechanical strength, which is mainly associated with the application of filaments made of technical polymers such as polyamide (PA) or polycarbonate (PC) (Ho et al. 1999). Commercial filaments, including those dyed in the mass with pigments based on inorganic compounds, may be associated with increased temperature-stable residue deposited in the mold cavity after the mold annealing process (Szadkowski et al. 2023).

Investment casting may be defined as an environmentally friendly, low-emission method, considering the energy balance compared to other foundry technologies (Prakash et al. 2021). However, the emissions generated as a result of the process (Prakash et al. 2021), although they have not been discussed before, may constitute a significant burden on the natural environment and a direct threat to people performing technological work, both in classic investment casting and its additive technology variant. Wojtyła et al. (2017) discussed VOC emissions arising during the FDM printing process. Despite processing filaments made of ABS, PLA, and PA at temperatures significantly below the initial temperature of polymer degradation, this process resulted in the release of potentially dangerous VOCs such as styrene, butanol, cyclohexanone, or ethylbenzene. In the case of investment casting, these materials are entirely degraded, and gases are uncontrollably released into the environment. This is the most significant disadvantage of using lost models made of polymers, as opposed to wax-based investment casting, where part of the modeling mass is or can be recovered and is not fully degraded.

This study aims to investigate emissions and environmental impact when using various thermoplastic polymers as models in investment casting technology. The screening qualitative chromatographic analysis of decomposition products sampled from various points of the production hall made it possible to define potential threats related to the processing of selected polymers and the necessary preventive measures. Furthermore, passive diffusion-type samplers were used at the sampling stage of VOCs emitted to the gaseous phase to reduce nuisance and user interference. Moreover, studies have been completed to characterize the used thermoplastic polymer degradation process. The coupled thermogravimetry (TGA) with analysis of gaseous products by infrared spectroscopy with Fourier transformation (FT-IR) has been used. The presented results are the first to compare and rate the use of this still-developing novel aspect of investment casting technology.

## Experimental

### Materials and sample preparation

**Fig. 1:**
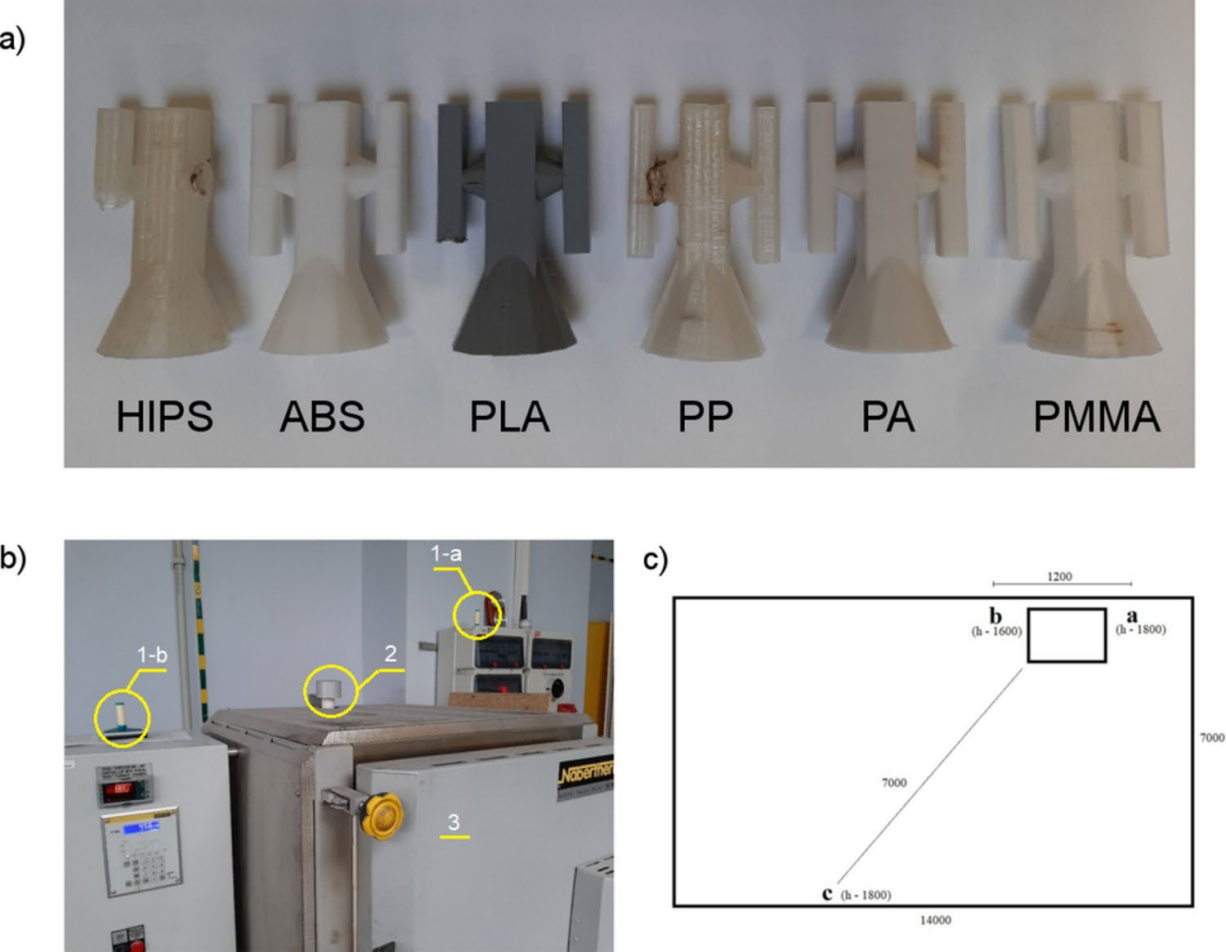
FDM-made models (**a**); photography of oven (3) with selected positions of measuring points of radiello^®^ diffusive passive samplers (1-a, 1-b) and air outlet from oven (2) (**b**); drawing with the geometry of sampling points in the technological hall (**c**)

The test samples were made of six types of polymers shaped using the FDM method using Zortrax ZM200 (HIPS, ABS, PLA, PP, PA) and three-dimensional printing (3DP) Voxeljet VX500 (PMMA) machines. The full description of the technical specifications of both 3D printers used is provided in the supporting information in Table S.1. The samples were made of commercially available filaments with a diameter of 1.75 mm as follows: high-impact polystyrene (HIPS) Filament HIPS Natural (Devil Design); acrylonitrile butadiene styrene terpolymer (ABS) Filament ABS + Natural (Devil Design); polylactide (PLA) Filament PLA Natural (Devil Design); polypropylene (PP) Fiberology PP Natural (Fiberology); polyamide 12 (PA) Filament PA12 Natural (Devil Design). The FDM models were created using the 0.4-mm nozzle. In the case of 3DP model manufacturing, the pol(methacrylate methacrylate) (PMMA) and poly(ethyl methacrylate) (PEMA) blend Solupor (Voxeljet) were delivered as a powder with an average particle size of 50 μm. The post-processing procedure includes infiltration by Voxeljet-Infiltrations-Wachs Typ A (Voxeljet) wax, based on hydrocarbon wax with a paraffin structure and melting point of 55 °C. Detailed parameters of the 3D printing process implemented in both technologies are provided in Table S.2 in supporting information. The procedure of sample preparation was related to those described by Polzin et al. (2013). The models are presented collectively in Fig. 1(a). In the next step, the samples were embedded in gypsum mass in steel cups, degassed for 5 min in a vacuum chamber, and left to solidify completely. The assembled plaster molds were placed in a Nabertherm 150 WAX oven at a temperature of 720 °C (Fig. 1(b)) to bake the polymer model. The location of the Radiello® passive samplers is shown in Fig. 1(b, c). The process was carried out for 4 h, followed by 8 h of airing the hall using an industrial exhaust system. Subsequent model firing processes were carried out in a 24-h cycle. A detailed description of the mold manufacturing process is included in the study (Kroma et al. 2020).

### Characterization

To collect the samples of VOCs emitted to the gaseous phase during the manufacturing process, the radiello^®^ diffusive passive samplers (*Fondazione Salvatore Maugeri*, *Padova*, *Italy*) were used. The selection of this type of sampling technique was caused by the fact that it does not disturb the users of the device in its regular exploitation. Additionally, due to the lack of power and gas supplies, it is possible to easily install passive samplers at almost every point in an indoor area. The mentioned passive sampler consists of three main elements: (i) a plastic triangular support with a clip to ensure the appropriate installation of the device; (ii) a microporous polyethylene (PE) yellow diffusion membrane (diffusion zone length, 150 mm; porosity, 10 ± 2 µm), and (iii) a cylindrical container made of steel net with sorption material—Carbograph 4 (300 ± 10 mg; 35–50 mesh) dedicated to collect VOC representatives and extract them from sorption medium using thermal desorption (TD) technique. More detailed data associated with the design and fields of application of mentioned diffusive passive samplers were described elsewhere (Zabiegała et al. 2010; Gallego et al. 2011; Król et al. 2012; Marć et al. 2014b). The exposure time of passive samplers in an indoor area was 6 h. There were three installed passive samplers inside the investigated enclosed area.

After the sampling period, the inlet of the radiello^®^ diffusive passive sampler containing sorption bed was removed and placed in the glass tube and closed with a PE cup. Next, vessels were transported to the laboratory to perform the final determination process. The extraction of VOCs retained on a sorption medium was performed using a two-stage thermal desorption technique (Unity Markes International, South Queensferry, UK) (Zabiegala et al. 2007; Marć et al. 2014a). Radiello^®^ inlets were placed inside a stainless steel tube and put inside the TD oven. Next, the tube was heated up to 290 °C for 15 min. Liberated organic compounds were transferred to the glass microtrap (0 °C) filled with two-type sorption medium (helium, 50 ml/min). After this, during the second stage of desorption, the microtrap was rapidly heated to 300 °C, and temperature was maintained for 5 min. Liberated analytes were directly transported in a splitless mode (helium, 1.0 mL/min) into the GC system (Agilent Technologies 6890) and separated on capillary column (30 m × 0.25 mm × 1 µm; J&W HP-1MS, USA). The TD-GC transfer line temperature was 160 °C. The oven program was as follows: 50 °C for 1 min, raised at a rate of 10 °C/min up to 120 °C maintained for 2 min, increased at the rate of 15 °C/min up to 280 °C and maintained for 5 min. The transfer line between the GC capillary column and mass spectrometer (5873 Network Mass Selective Detector, working mode—SCAN) was 280 °C. The mass spectrometer (MS) ionization source (IS) temperature was set to 230 °C, and the quadrupole temperature was 150 °C. Identification and qualitative analysis of emitted VOCs were performed using the mass spectra database (NIST 2.0 Mass Spectral Library) attached to the MS system software (The NIST Mass Spectral Search Program for the NIST/EPA/NIH Mass Spectral Library Version 2.0 d, USA). For further data interpretation, only the degree of correspondence between the spectrum of the compound and the spectrum from the database above 85% was considered.

The decomposition process of tested materials was carried out on a thermogravimetric analyzer (NETZSCH STA) coupled to an infrared spectrometer (TENSOR 27 FT-IR) to analyze evolved gases. Samples of around 10 mg were conducted under an air atmosphere (30 ml/min) using a thermogravimetric analyzer. The temperature ranged from 30 to 900 °C with a heating rate of 10 °C/min. Gas products from STA were output to the FT-IR spectrometer through a heated up to 230 °C transfer line. The FT-IR gas cell was also heated to 200 °C to prevent the condensation of volatile products.

## Results and discussion

### VOCs’ emissions during materials processing

Analysis of the VOCs’ emissions is an essential part of assessing processes’ safety. Moreover, it is crucial for the development of genuinely environmentally friendly processes. However, it is rather rarely investigated and described in the literature. Developing sustainable and environmentally friendly processes requires a comprehensive evaluation of environmental impacts. Therefore, during the performed thermal removal of models, passive dosimetry was applied to collect generated VOCs and analyze them using chromatographic techniques.

**Fig. 2:**
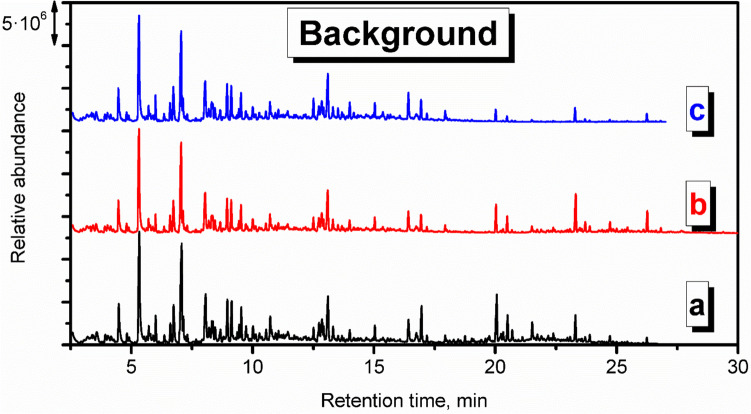
Chromatogram of the VOCs emitted by the annealing of gypsum mold used for definition of the surrounding environment during analysis of the mold preparation in investment casting (letters a, b, and c indicate positions of measuring points of radiello® diffusive passive samplers presented in Fig. 1)

Nevertheless, it is crucial to remember that VOCs are present in the surrounding indoor and outdoor environment due to the typical applications of various man-made materials, as well as the emissions from plants and other natural materials. Therefore, precise assessment of VOCs’ emissions during the process requires analysis of the background, i.e., the VOCs present when the investigated process is not conducted. In the presented case, the goal was to assess the emissions originating from applying particular polymer materials during the investment casting procedure. Therefore, the background analysis was performed for the thermal treatment of the gypsum mold without the 3D-printed model inserted into the gypsum mold. The results of the performed analysis are presented in Fig. 2 in the form of a chromatogram, and Table 1 lists the detected compounds and provides the data related to the hazards they pose to human health and the environment. To be precise, not all detected compounds should be called VOCs. However, there is no commonly agreed, straightforward definition of volatile organic compound and limit of vapor pressure. Taking into account different interpretations, VOC should be characterized by a boiling point below 250 °C or vapor pressure exceeding 13 Pa. Therefore, among the detected compounds listed in Table 1, only higher-molecular weight hydrocarbons (C_13_ and more) should not be considered VOCs straightforwardly.

**Table 1:**
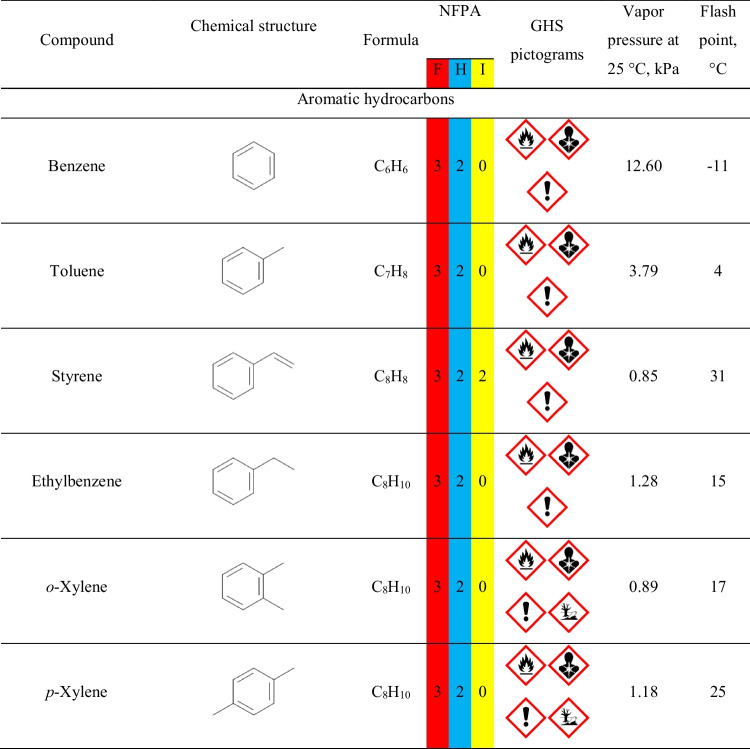

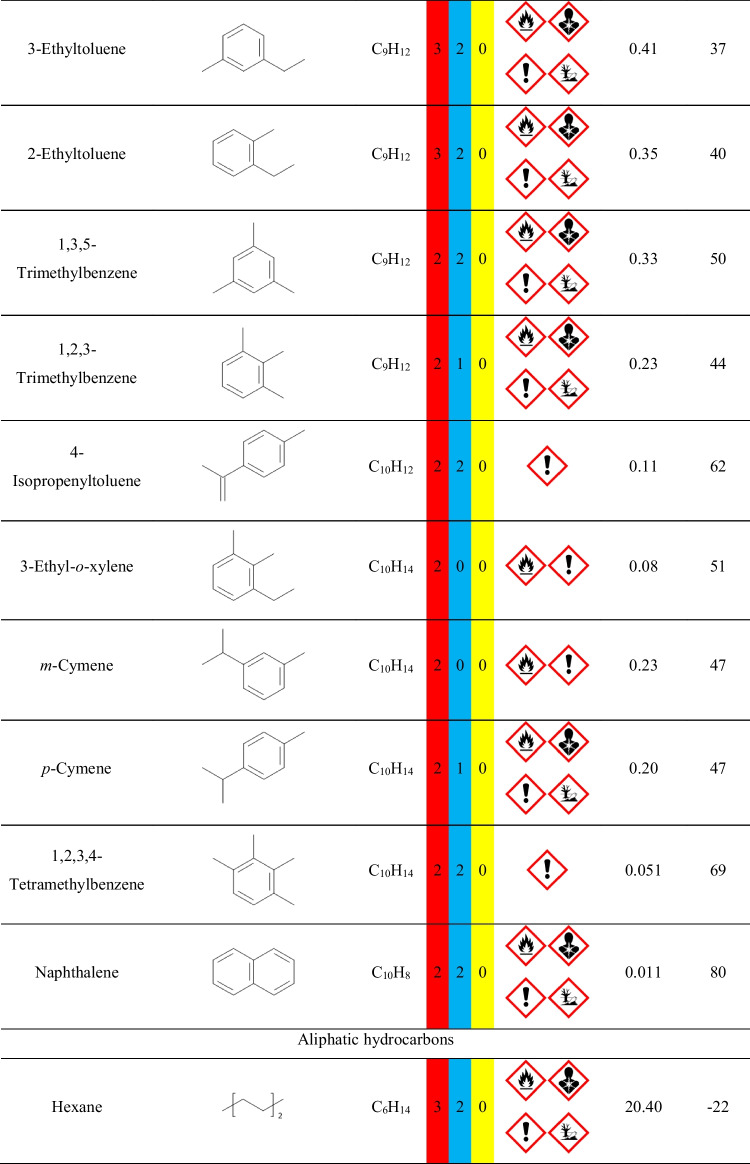

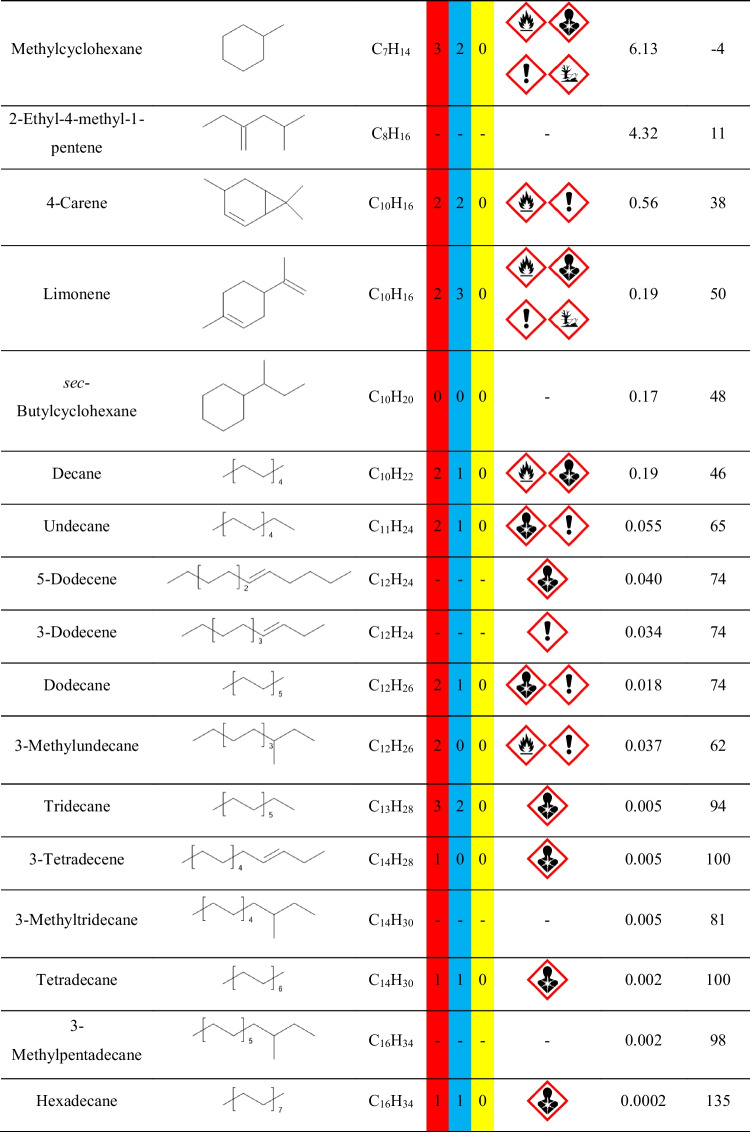

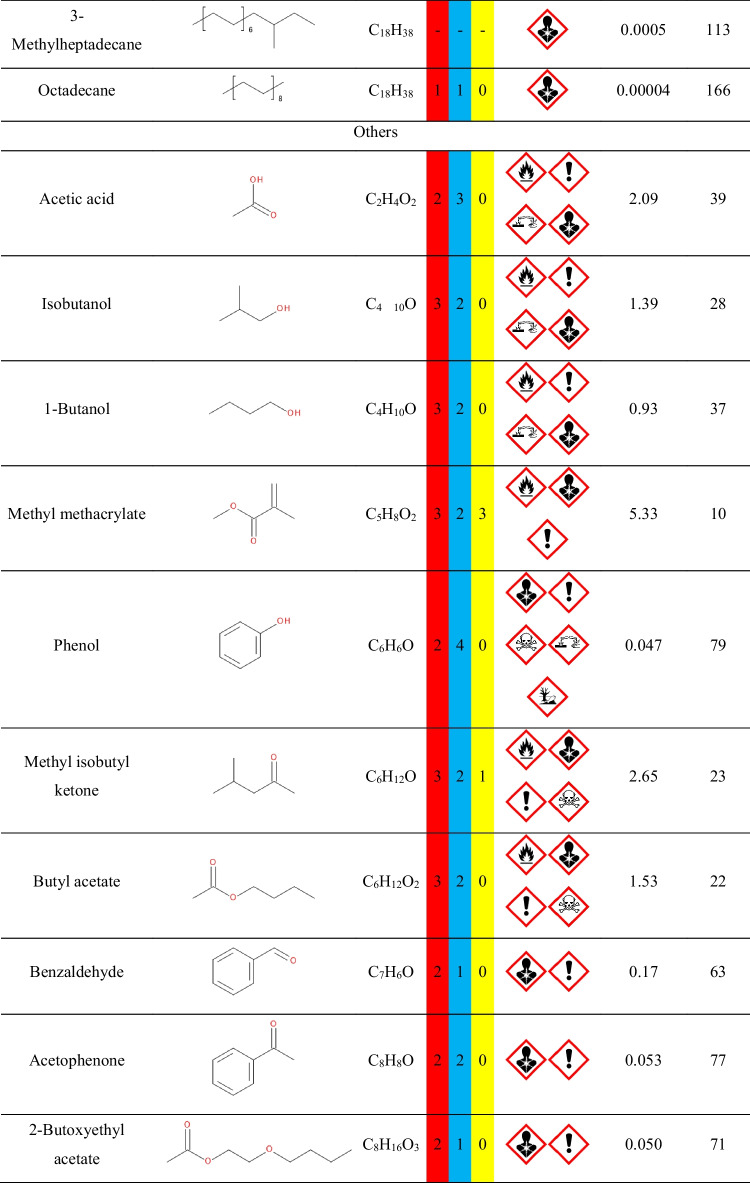
List of compounds in the surrounding environment

The presence of compounds listed in Table 1 in the surrounding environment may be attributed to the other analysis and processes conducted in the same compartment, yielding the adsorption of VOCs by multiple surfaces, as well as to the emissions from building and finishing materials applied in the analyzed compartment, as reported in our previous works (Hejna et al. 2021c, b, a).

For example, the presence of aromatic hydrocarbons can be associated with the emissions from vinyl floorings and tiles, synthetic rubber parts, polyurethane foams, and materials from polystyrene or polyester resins (Nielsen et al. 1994; Yu and Crump 1998; Hillier et al. 2003). On the other hand, aliphatic hydrocarbons have been repeatedly detected among the VOCs emitted from polyethylene, polypropylene, or natural rubber products (Willoughby et al. 2003; Hoven et al. 2003; Curran et al. 2016). Terpenes and terpenoids included in these two groups of compounds may have originated from natural plant-based products, including wood-based materials (Ståhl et al. 2004; Tsuji and Mizuno 2010). Considering the rest of VOCs, collectively named Others, their origin is also attributed to various commonly applied synthetic materials. Acetic acid has been detected among VOCs emitted from natural rubber and polyolefins (Willoughby et al. 2003; Hoven et al. 2003; Curran et al. 2016). Methyl methacrylate can be found in poly(methyl methacrylate) as the residual monomer or generated during this polymer’s aging (Tsai et al. 2009; Curran et al. 2016). Phenol has been reported to be emitted by floorings and carpets made from poly(vinyl chloride), polystyrene, or phenol formaldehyde resins (Wolkoff 1998; Yu and Crump 1998). Methyl isobutyl ketone is applied in the rubber industry and has been detected among VOCs emitted from rubber products (Sakai et al. 2022; Hejna et al. 2023). Butyl acetate is commonly applied when producing various polymeric coatings (Yu and Crump 1998). Benzaldehyde, similar to acetophenone, is often detected due to the oxidation of styrenic units in polystyrene or polyester resins (Lattuati-Derieux et al. 2013).

In summary, the presence of the abovementioned VOCs in the gas phase inside the laboratory hall, finished with polymer-based building materials, where plastics are commonly processed and analyzed, is fully justified.

However, the aim of the presented work was not only to determine VOCs emitted during the thermal removal of models as a step of investment casting but also to assess the impact of the process on the surrounding environment and human health. Therefore, Tables S.3 and S.4 provide details on the safety data for detected compounds based on two internationally accepted systems. The first one is maintained by the U.S.-based National Fire Protection Association. It characterizes chemical compounds with color-coded graphics commonly known as safety square or fire diamond. The square-shaped graphic is divided into four separate squares representing flammability, health hazards, instability-reactivity, and special notices. The first three squares are colored respectively red, blue, and yellow and include a proper number, from 0 to 4, which quantifies the hazards. The fourth square is white and optionally includes special symbols for strong oxidizers, water-reactive compounds, or asphyxiant gases.

The second system is the Globally Harmonized System of Classification and Labelling of Chemicals (GHS), often applied to mark chemicals according to the hazards posed to human health and safety. The GHS specifies the particular properties and their values, which are used to determine the threats posed by various chemicals. It includes physical, health, and environmental hazards to humans and the environment. The information based on GHS is often included in safety datasheets of chemicals. For simplification, it describes chemicals with a combination of hazard statements and pictograms. Table S.4 describes the nine pictograms used by GHS, along with the corresponding statements. The attribution of hazard individual statements to specific compounds is more complicated than the NFPA standard due to the multiplicity of described hazards and their grading.

Except for the hazard information, Table 1 provides information on the vapor pressure and flash point values of detected compounds, which are also part of the NFPA and GHS assessment. As mentioned above, the value of vapor pressure is often taken into account during the classification of the compound as volatile, as it quantifies the liquid’s thermodynamic tendency to evaporate, described as the balance of particles escaping from the liquid (or solid) in equilibrium with those in a coexisting vapor phase. The second parameter, flash point, is described by the EN 60079–10-1 standard as the lowest liquid temperature at which, under certain standardized conditions, a liquid gives off vapors in quantity such as to be capable of forming an ignitable vapor/air mixture.

It can be seen that multiple detected compounds show vapor pressure exceeding 2.3 kPa, which is the value for water in the air at sea level and saturated with water vapor at 20 °C. Therefore, such values indicate significant volatility, resulting in significant health hazards, as indicated by the NFPA and GHS rankings.

**Fig. 3:**
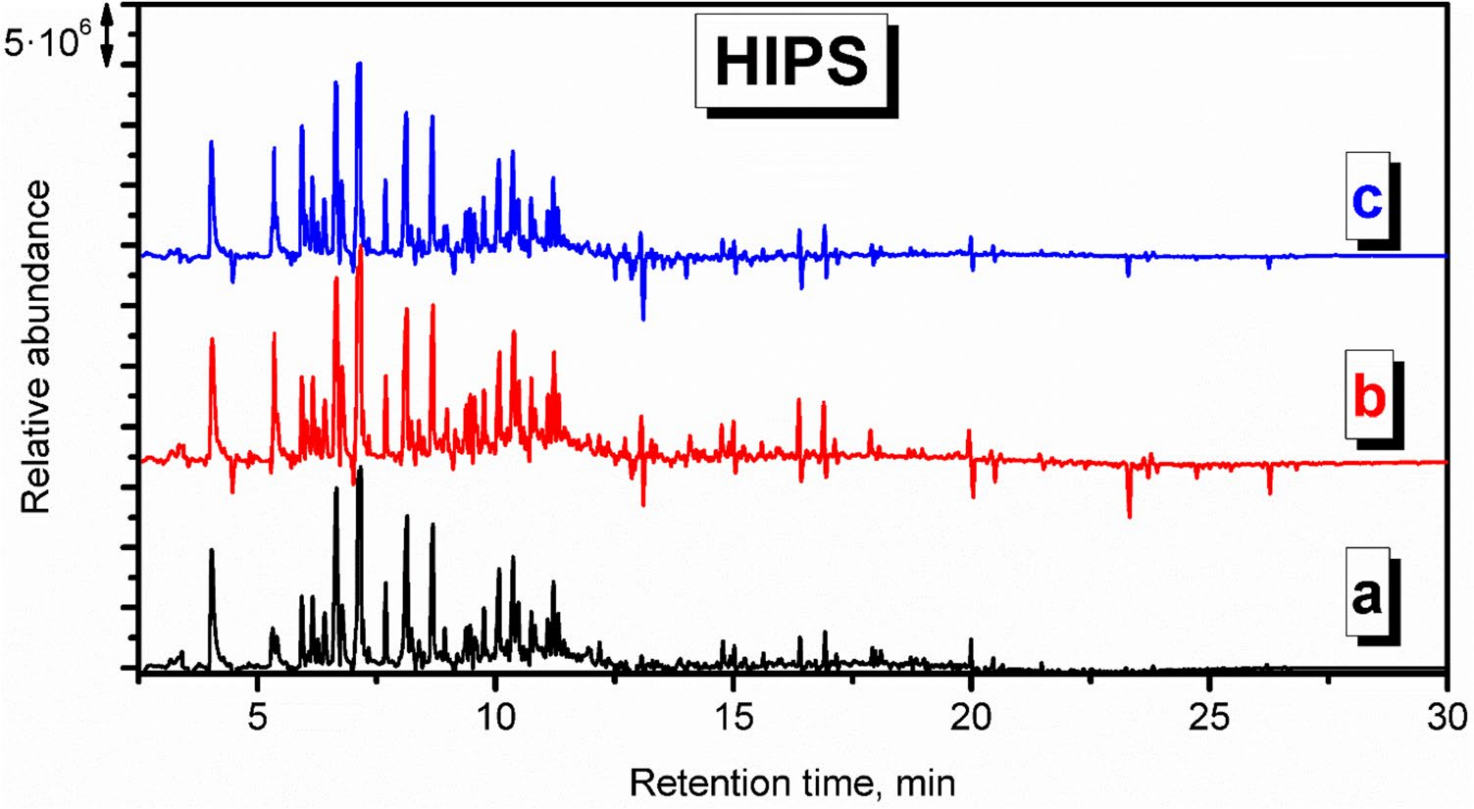
Derivative chromatogram of the VOCs emitted from thermal degradation of HIPS during the procedure of mold preparation in investment casting

After assessing the background emissions in the analyzed compartment, the emission profiles from particular samples can be determined. Fig. S.1 shows the chromatogram obtained during the analysis of VOCs collected for thermal removal of the HIPS model, while Fig. 3 presents the derivative chromatogram resulting from the subtraction of the background chromatogram from the one obtained during the analysis of VOCs’ emissions related to HIPS’s processing.

It can be seen that even after subtraction of background data, multiple VOCs have been detected, especially for retention times below 12 min, which points to the multitude of low-molecular-weight compounds. The most abundant VOC was styrene, which is a monomer applied for HIPS production and is always detected among the VOCs emitted from various types of PS, either at ambient temperatures, during processing or thermal decomposition (Jakab et al. 2003; Vilaplana et al. 2010; Yuzawa et al. 2013). Thermally induced chain scissions occurring during the model thermal removal process also yielded the presence of benzene, toluene, ethylbenzene, 1-methylethyl benzene, α-methyl styrene, *o*-isopropenyltoluene, naphthalene, and xylenes, all detected in significantly higher amounts compared to the background check (Yuzawa et al. 2013). The evolution of branched or polyaromatic derivatives can be associated with the dimerization or trimerization and further cyclization and dehydrogenation reactions of monomer styrene units (Jakab et al. 2003). On the other hand, due to the vulnerability of HIPS to oxidation related to the presence of unsaturated bonds, both in the PS phase and in polybutadiene rubber applied as a modifier, oxidized benzene derivatives were detected—benzaldehyde, benzofuran, phenol, and acetophenone (Rezig et al. 2020). Moreover, the decomposition of the PB phase may yield the saturated and unsaturated aliphatic hydrocarbons, among which the most abundant were 2,3-dimethyl heptane, 2,4-dimethyl-1-heptene, 2,6-dimethyl nonane, and mostly 3,7-dimethyl decane (Vilaplana et al. 2010; Lattuati-Derieux et al. 2013).

**Fig. 4:**
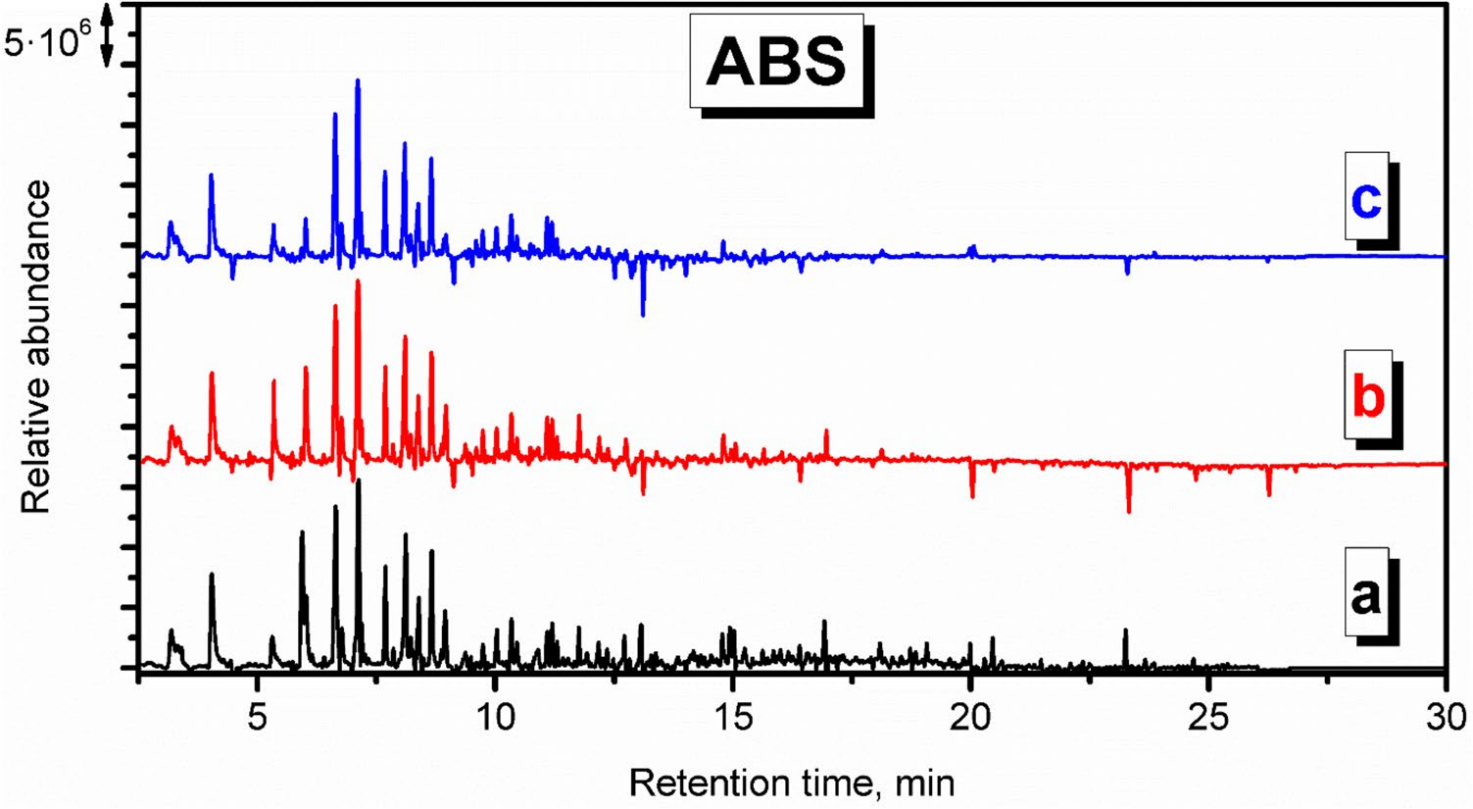
Derivative chromatogram of the VOCs emitted from thermal degradation of ABS during the procedure of mold preparation in investment casting

**Fig. 5:**
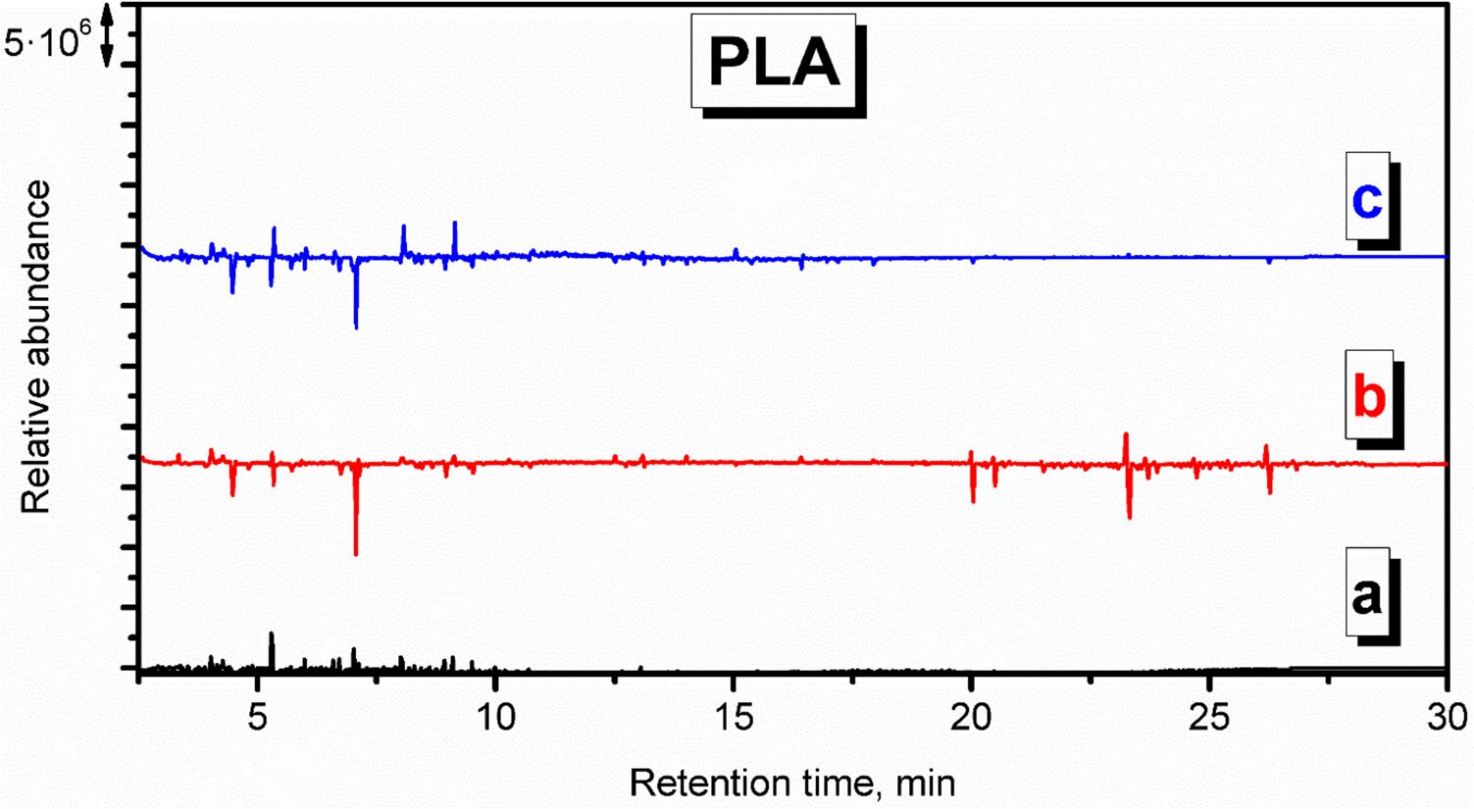
Derivative chromatogram of the VOCs emitted from thermal degradation of PLA during the procedure of mold preparation in investment casting

**Fig. 6:**
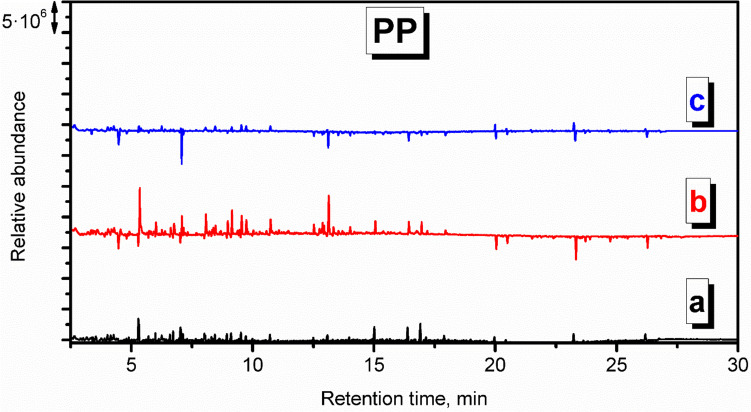
Derivative chromatogram of the VOCs emitted from thermal degradation of PP during procedure of mold preparation in investment casting

**Fig. 7:**
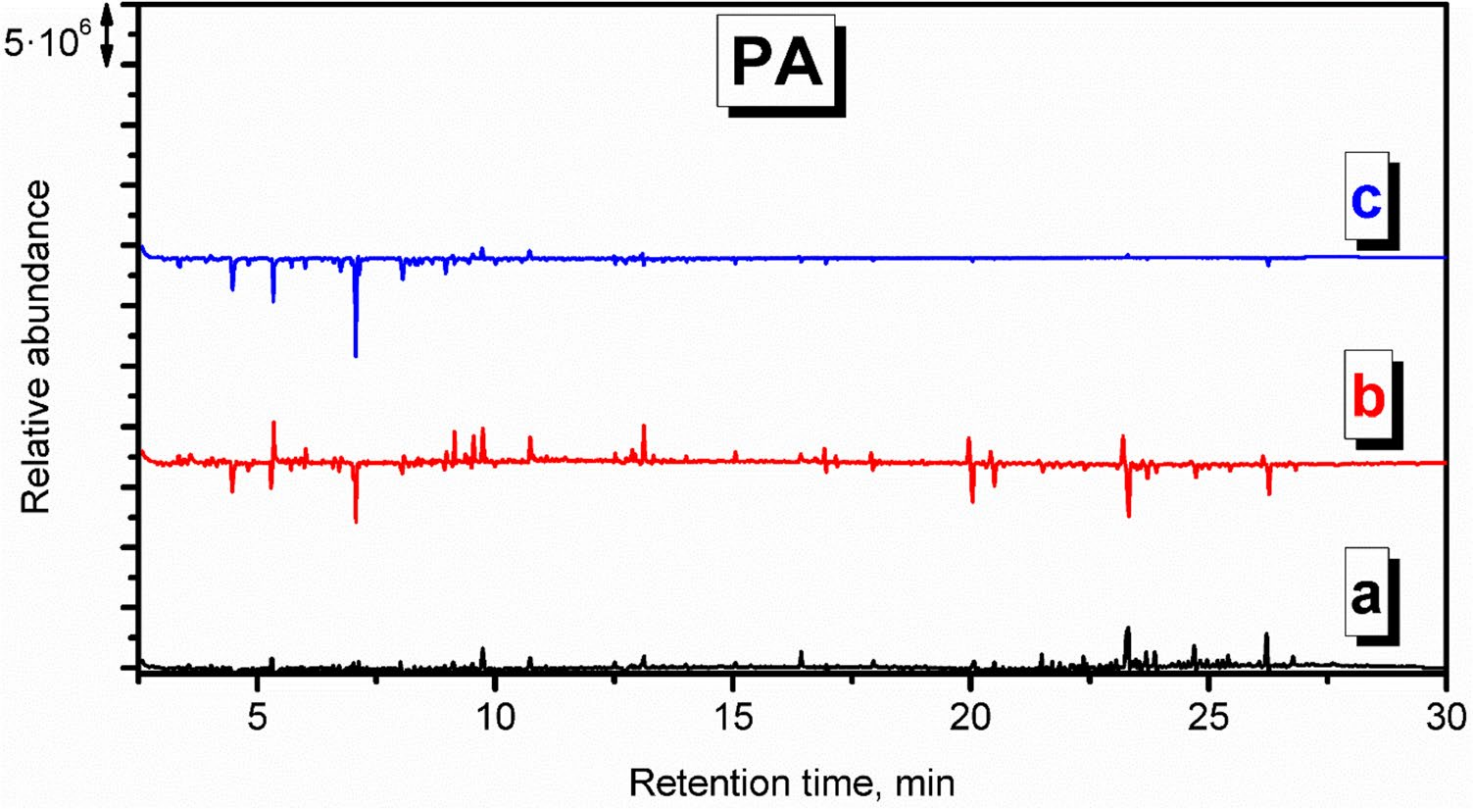
Derivative chromatogram of the VOCs emitted from thermal degradation of PA during procedure of mold preparation in investment casting

**Fig. 8:**
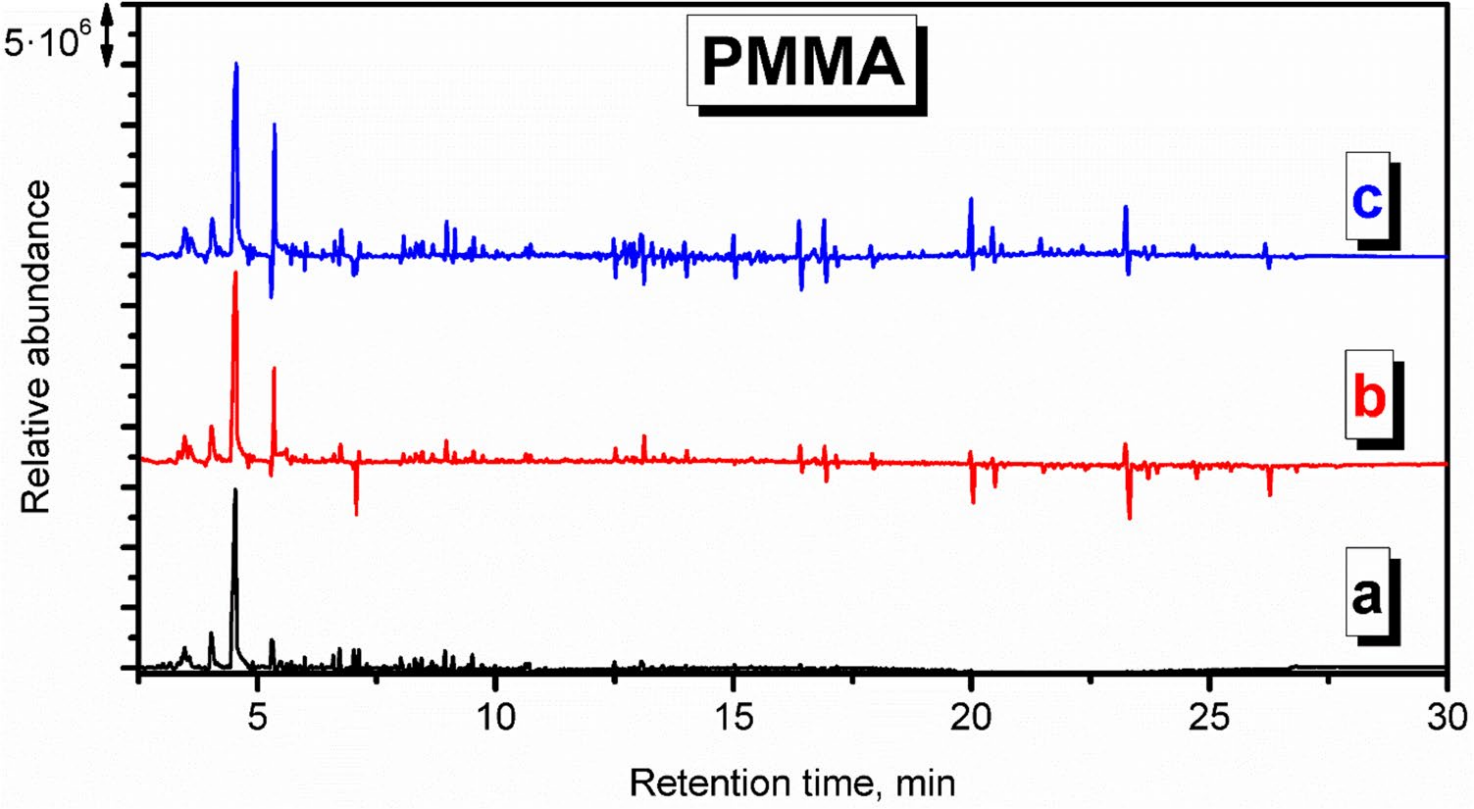
Derivative chromatogram of the VOCs emitted from thermal degradation of PMMA during the procedure of mold preparation in investment casting

Considering the structural similarities related to the presence of styrenic and butadiene units, the chromatogram depicted for VOCs’ emissions from ABS was relatively similar to the HIPS, as presented in Figs. S.2 and 4. The most significant difference was attributed to the presence of benzonitrile (~ 8.4 min), which has already been detected among ABS thermal decomposition products (Saraji-Bozorgzad et al. 2011), and reduced emissions for retention times exceeding 9.3 min. The latter effect was associated with the limited emissions of decane and dodecane derivatives, which, in the case of HIPS, could be related to the decomposition of the rubber modifier. Other, less noticeable differences were associated with the presence of 2-methylpentane among the butadiene degradation products and the presence of biphenyl and naphthalene derivatives, pointing to the more pronounced dimerization of aromatic moieties (Figs. 4, 5, 6, 7 and 8).

Figures S.3 and 5 show the chromatograms for the VOCs detected during the PLA application in the model thermal removal process. Contrary to HIPS and ABS, the appearance of the virgin chromatogram is noticeably different, similar to the background check, and the number of signals is lowered, which can be explicitly seen in the derivative plot. Among the compounds characteristic of PLA can be mentioned acetic acid, repeatedly reported among VOCs emitted from PLA during 3D printing (Davis et al. 2019; Wojnowski et al. 2022). Moreover, various esters of carboxyl acids were detected, like hexyl isovalerate, butyl acetate, and 2-butoxyethyl acetate, which also confirms the literature data (Davis et al. 2019). Interestingly, the most abundant VOCs detected at sampling point c were toluene, benzaldehyde, and decane. They were, however, hardly detected closer to the oven, which points to their background origin.

Figures S.4 and 6 present the chromatogram recorded for the application of PP. Similar to PLA, the appearance of the chromatogram is very similar to the background check, pointing to the small amounts of emitted VOCs and efficient decomposition of PP material. At sampling point c, which was located 7 m from the oven, hardly any VOCs have been detected. The magnitude of signals related to the presence of aliphatic hydrocarbons was enhanced, including decane, undecane, 3-dodecene, dodecane, tridecane, 3-methyltridecane, or tetradecane. These compounds are typically detected from PP (Willoughby et al. 2003).

Chromatograms indicative of PA emissions (Figs. S.5 and 7) point to the low magnitude of signals suggesting limited emissions. Holland and Hay suggested that the chemical changes during thermal degradation include crosslinking, the formation of an aromatic-conjugated material, and, ultimately, an involatile black char, which may explain the low level of detected VOCs (Holland and Hay 2000). Such a phenomenon may explain the reduced amounts of detected styrene, xylenes, and benzaldehyde compared to the background check. Moreover, the lack of nitrogen-containing compounds among detected VOCs suggests their low molecular weight, below the detection level, or their involvement in the crosslinking reactions (Holland and Hay 2000).

The decomposition of PMMA resulted mainly in the emissions of methyl methacrylate, as presented in Figs. S.6 and 8. Depolymerization is a typical mechanism of PMMA decomposition (Holland and Hay 2002). Moreover, a significant amount of aromatics were detected, including benzene, toluene, xylenes, and benzene derivatives (Holland and Hay 2001). Applied PMMA material also contained paraffin as an additive facilitating processing, which also affected the VOCs’ emissions. Signals noted at longer retention times point to the presence of longer aliphatic hydrocarbons related to the paraffin decomposition, including 3-methylpentadecane, hexadecane, 3-hexadecene, 3-methylheptadecane, heptadecane, octadecane, or 3-octadecene.

**Fig. 9:**
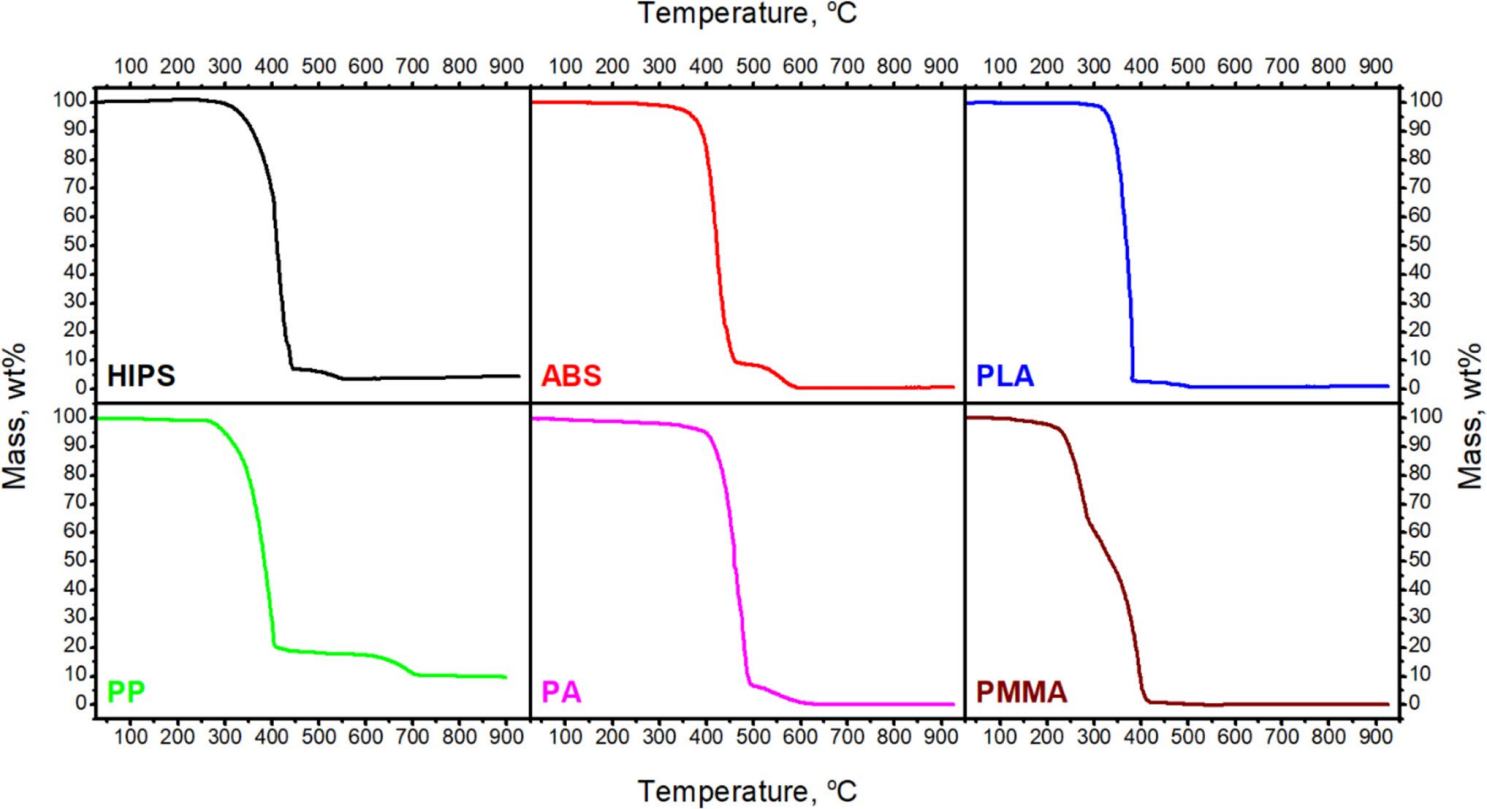
TG curves of filaments used during the studies

**Fig. 10:**
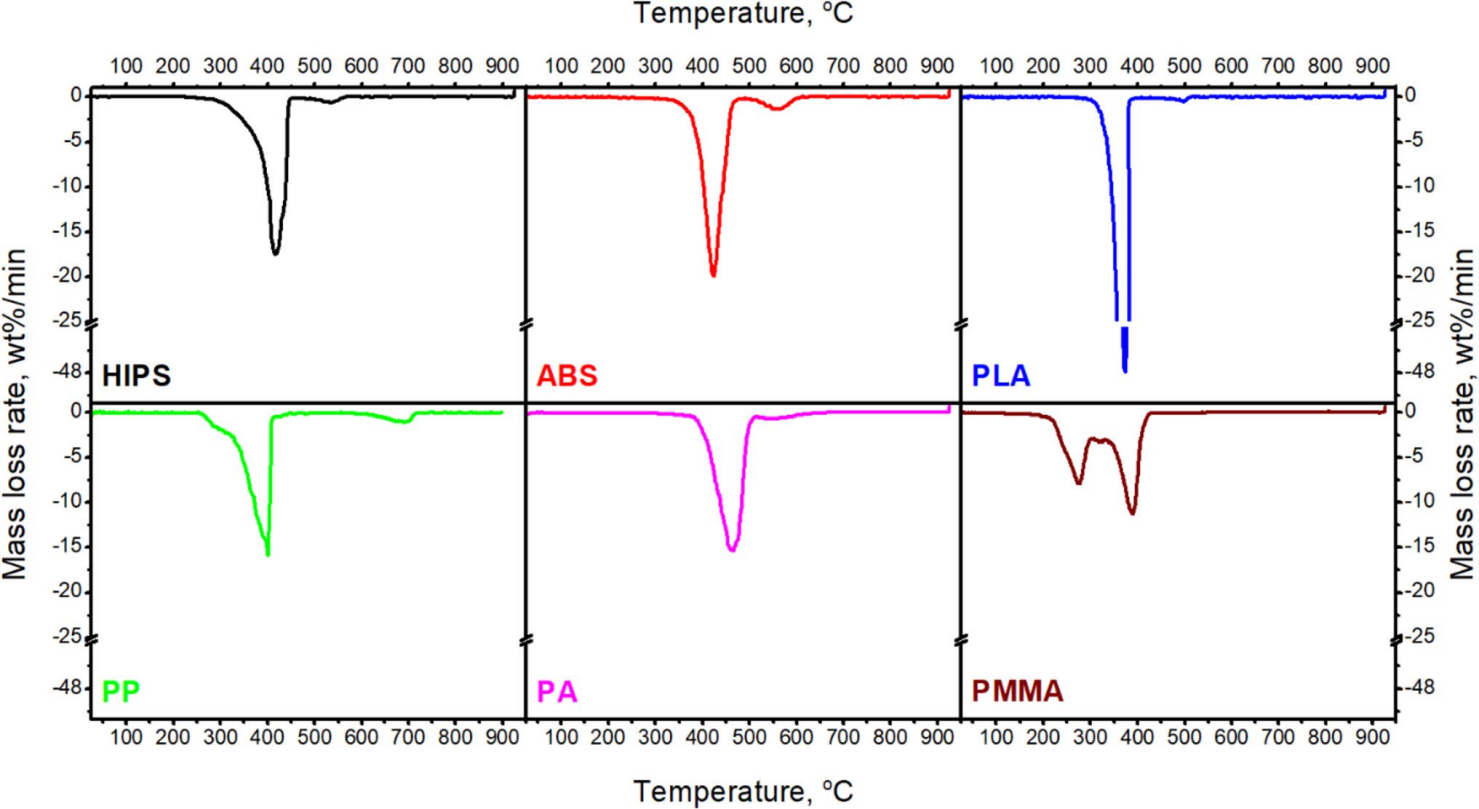
DTG curves of filaments used during the studies

**Fig. 11:**
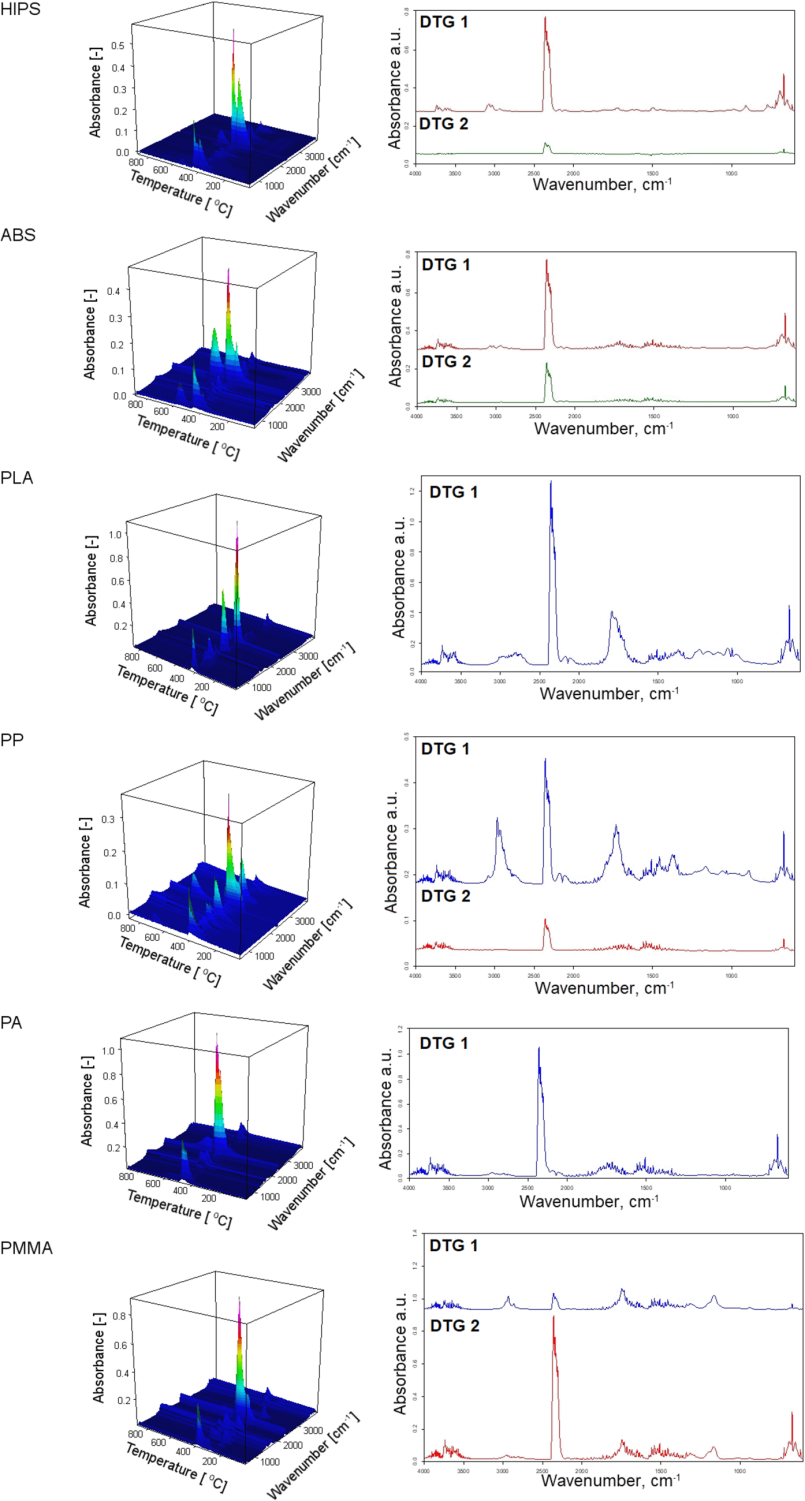
FT-IR spectra of gaseous products generated during thermal decomposition as a function of temperature, and FT-IR spectra taken at DTG peaks measured

The emissions during the thermal decomposition of FDM molded models are related to the thermal decomposition of applied polymers. An additional detailed investigation using thermogravimetric analysis coupled with spectroscopic analysis of the gaseous products has been undertaken. The mass (TG) and derivative mass loss (DTG) curves of all tested materials are shown in Figs. 9 and 10. From the TG curve, the temperature of 5% of mass loss (*T*_5%_), 10% of mass loss (*T*_10%_), 50% of mass loss (*T*_50%_), and residues at 900 °C (*R*_900_) were determined. From the DTG curve, the temperature of maximum decomposition peaks (*T*_max 1_ and *T*_max 2_) and the maximum mass loss rate were determined. The characteristic temperatures are summarized in Table 2. The FT-IR spectra were determined at the temperature connected with the maximum mass loss peak. Significant components like CO_2_ (2400–2300 and 670 cm^−1^), H_2_O (4000–3500 and 2000–1500 cm^−1^), and CO (2200–2100 cm^−1^) at the FT-IR spectra were observed for all tested materials. The FT-IR spectra obtained during the decomposition process are presented collectively in Fig. 11. The 3D images of changes in absorption spectra as a function of temperature in the heating chamber have been supplemented with FT-IR spectra taken at characteristic temperature values of DTG peaks for each polymer.

**Table 2:** TG and DTG data of testing polymers

Materials	*T*_5%_, °C	*T*_10%_, °C	*T*_50%_, °C	DTG 1, °C; %/min	DTG 2, °C; %/min	*R*_900_, %
HIPS	339	359	411	417; 17.47	534; 0.63	4.4
ABS	371	390	422	423; 19.93	561; 1.37	0.6
PLA_grey	330	340	371	375; 70.70	-	0.1
PP	299	322	384	401; 15.80	691; 1.09	9.7
PA	397	417	460	464; 15.34	-	0.1
PMMA with paraffin	231	247	336	275; 7.92	389; 11.20	0.1

The HIPS material has two stages of degradation at 417 °C and 534 °C with a maximum mass loss rate of 17.47%/min and 0.63%/min, respectively, which are observed. The first stage is attributed to the formation of toluene and ethylbenzene bands (2800, 1596, and 1721 cm^−1^) and styrene moieties (3069 and 1596 cm^−1^) (Xianfeng et al. 2019). In the second stage, all signals disappear. Exceptions are peaks connected with CO_2_ and CO bands. Relatively high char residue is observed, probably due to the presence of synthetic rubber used to produce HIPS. In the case of ABS, two stages of degradation were also observed. At the first one at 422 °C, the maximum mass loss rate is 19.93%/min. At this stage, the FT-IR spectra of the evolving gases, except for gases present in all materials, also show small peaks connected to the aliphatic (butadiene; 2850–2970 cm^−1^) and aromatic (styrene; 3010–3110 cm^−1^) vibrations (Du et al. 2011). At the second stage (561 °C), with a maximum mass loss rate of 1.37%/min, the peaks from the aromatic and aliphatic bands disappear, and the intensity of other bands decreases. The PLA shows a one-step mass loss with the maximum at 374 °C. The FT-IR spectra show the occurrence of peaks attributed to the asymmetric vibrations of the ester group, asymmetric bending of the CH_3_ group, and the carbonyl stretching (1183 cm^−1^, 1457 cm^−1^, and 1750 cm^−1^, respectively) (Jauzein et al. 2017; Cuadri and Martín-Alfonso 2018). The PP material has two stages of decomposition at 401 °C and 691 °C. At the first decomposition step, the peaks connected with unsaturated hydrocarbons (2750–3100 cm^−1^) and signals connected with branched chains (C–H, C = C, C–C) are observed. At the second stage of decomposition, only CO_2_ and CO are observed. The highest char residue was observed for this material. The additional decomposition step at 691 °C observed in the TG and DTG curves is related to the addition of calcium carbonate used as a filler and its degradation. Calcium carbonate is often used as a functional filler for polypropylene due to its thermodynamic stability. The observed range of filler decomposition temperature corresponds to the data described in the literature (Li et al. 2017; Karunadasa et al. 2019). In the case of PA, one decomposition step at 464 °C was observed (Table 2). At this stage, FT-IR spectra show signals connected with unsaturated hydrocarbons (2750–3100 cm^−1^), and signals connected with branched chains (C–H, C = C, C–C) and CO_2_ and CO are observed. In the wavenumber range 950 cm^−1^ and 1460–1600 cm^−1^, peaks of low absorbance are visible, which could indicate the presence of ammonia (Von et al. 2012). These peaks overlap with peaks attributed to H_2_O. This material has the best thermal stability, about what proves the highest *T*_5%_. For PMMA with paraffin, two decomposition steps at 275 °C and 389 °C were observed. The first one is connected with the decomposition of paraffin (Zhang et al. 2020). At this stage, a functional group such as C–H (bands at 2931 cm^−1^), C = O (bands at 1741 cm^−1^), and CH_2_ (bands at 1470 cm^−1^) are shown. The second decomposition step is attributed to the decomposition of PMMA (Yang et al. 2018). The primary decomposition products are propionic acid and methyl ester (Alghunaim 2015). In the range of 310–340 °C, the little peaks at the DTG curve are observed. They are connected with the paraffin. In this range of temperatures, paraffin has the fastest mass loss (Xianfeng et al. 2019).

## Conclusions and future remarks

This study addresses the issue of determining environmental hazards and emissions of VOCs generated during the production of molds using 3D printing models for investment casting technology. Commonly used commercial 3D printing materials were analyzed, including five types of thermoplastics used to shape models using fused deposition modeling (FDM), including polylactide (PLA), acrylonitrile butadiene styrene terpolymer (ABS), high-impact polystyrene (HIPS), polyamide 12 (PA12), and polypropylene (PP) and one poly(methyl methacrylate) (PMMA) grade formed in the three-dimensional printing (3DP) process. Following the obtained results, apparent differences were observed in the VOC emission profile depending on the type of thermoplastic polymers used as models in investment casting technology. The emission profile and emission intensity of VOCs directly rely on the kind of printing materials used. The research results were supplemented with thermal analysis coupled with the assessment of gases released during decomposition by infrared spectroscopy with Fourier transform (FTIR). Research has shown that despite the investment casting technology coupled with the production of models using the FDM method being considered a relatively low-emission technology, special attention should be paid to the protection of technical staff and the protection of technological exhaust gases, limiting uncontrolled environmental emissions. Based on the presented results, there are two main directions, which can be interchangeably used, depending on the desired performance of the printed part.

The first one assumes the application of styrenic or acrylate polymers like ABS, HIPS, or PMMA, which require particular caution and preventive measures. It can be addressed by the application of efficient filters or adsorbents, limiting the release of particularly hazardous VOCs to the environment. However, such solutions are often selective towards particular compounds, which may be limited when structurally different compounds are emitted during the process. It is important to remember that polymers are composed of high-molecular-weight macromolecules, whose chemical structure and spatial arrangement are often within a certain range, so the particular batches may slightly differ, which could affect the decomposition mechanism and the structure of generated VOCs, e.g., by the release of styrene derivatives instead of styrene.

The other direction is the application of low-emission materials, which would not require additional safety measures. The presented results clearly indicated that PLA and PA are among such materials. They are widely used in 3D printing, so their application in the described process is relatively straightforward. Moreover, they yield printed parts with performance, enabling efficient use in investment casting. Thermogravimetric analysis indicated that they could be almost entirely removed from the mold during the process, which is their additional advantage. In terms of PLA, the unprecedented benefit is also their renewable origin, which limits the environmental burdens. In the future, it will also be interesting to apply bio-based PA grades, which could enhance environmental friendliness compared to the conventional PA 12 grade.

## Supplementary information

Below is the link to the electronic supplementary material.

Supplementary file1 (DOCX 1155 KB)

## Data Availability

The datasets generated during and/or analyzed during the current study are available from the corresponding author on reasonable request.
