# Supplementary material for: Analysis of emission of volatile organic compounds and thermal degradation in investment casting using fused deposition modeling (FDM) and three-dimensional printing (3DP) made of various thermoplastic polymers
Source: Environ Sci Pollut Res Int. 2024 Oct 9;31(50):60371–88. doi: 10.1007/s11356-024-35200-x (PMC11519180; doi:10.1007/s11356-024-35200-x)
Supplement: Supplementary file 1 — Supplementary file1 (DOCX 1155 KB) [file 11356_2024_35200_MOESM1_ESM.docx]

**Analysis of emission of volatile organic compounds and thermal degradation in investment casting using fused deposition modeling (FDM) and three dimensional printing (3DP) made of various thermoplastic polymers**

**SUPPORTING INFORMATION**

**Table S.1.** Technical details about 3D printers used for models manufacturing

| **Technical data** | **Value** |
| --- | --- |
| 3D printer | **Zortrax M200** |
| Available processes and materials | LPD (Layer Plastic Deposition) – depositing melted material layer by layer onto the build platform; thermoplastics in form of 1.75 mm filament. |
| Build space | 200x200x180 mm |
| Nozzle diameter | 0.4 mm |
| Layer resolution | 90-390 μm |
| Minimal wall thickness | 450 μm |
| Platform Levelling | Automatic measurement of platform points’ height |
| Maximum temperature | 290 °C |
| Build Platform | \|  \| Heated \| \| --- \| --- \| |
| Maximum Platform Temperature | 105 °C |
| Ambient Operating Temperature | 20-30 °C |
|  | |
| 3D printer | **VX500 Voxeljet** |
| Available processes and materials | - sand (with furan / phenolic adhesive)  - PMMA  - ceramic |
| Build space LxWxH | 500x400x300 mm |
| Build speed | 15 mm/h / 3 l/h |
| Print resolution (X, Y, Z) | 600 dpi |
| Layer thickness | 150 μm |
| Head type | Multi-jet |

**Table S.2. Printing parameters used for manufacturing**

| **Material** | **Extrusion temperature** | **Platform temperature** | **Layer height** | **Retraction** | **Infill degree** |
| --- | --- | --- | --- | --- | --- |
|  | [°C] | | [μm] | [mm/s] | [%] |
| PLA | 207 | 30 | 140 | 36 | 10 |
| ABS | 275 | 80 | 140 | 36 | 10 |
| HIPS | 255 | 80 | 140 | 30 | 10 |
| PA11 | 250 | 70 | 140 | 30 | 10 |
| PP | 250 | 80 | 140 | 36 | 10 |
| **Material** | **Process temperature** | | **Temperature of paraffin impregnation** | | |
|  | [°C] | | | | |
| PMMA | 25 | | 70 | | |

**Table S.3.** Description of the ratings used in the NFPA 704: Standard System for the Identification of the Hazards of Materials for Emergency Response.

| Flammability | |
| --- | --- |
| Rating | Description |
| 0 | Noncombustible materials like stones, sand, or concrete, which will not burn in the air unless exposed to a temperature of 820 °C for more than 5 minutes. |
| 1 | Materials requiring significant preheating before ignition and combustion, characterized by the flash point at or above 93.3 °C. |
| 2 | Materials igniting after moderate heating or exposure to relatively high ambient temperature, characterized by the flash point between 37.8 and 93.3 °C. |
| 3 | Liquids and solids possibly ignited under almost all ambient temperature conditions, characterized by the flash point between 22.8 and 37.8 °C or by the combination of the flash point below 22.8 °C and boiling point at or above 37.8 °C. |
| 4 | Compounds rapidly or completely vaporizing at normal atmospheric pressure and temperature, readily dispersing in air and igniting readily, characterized by the flash point below 22.8 °C. |
| Health hazards | |
| Rating | Description |
| 0 | Compounds posing no health hazard, requiring no precautions and offering no hazard beyond that of ordinary combustible materials. |
| 1 | Materials irritating with only minor residual injury after exposure. |
| 2 | Materials causing temporary incapacitation or a possible residual injury after intense or continued but not chronic exposure. |
| 3 | Materials causing serious temporary or moderate residual injury after short exposure. |
| 4 | Materials causing death or a major residual injury after very short exposure. |
| Instability-reactivity | |
| Rating | Description |
| 0 | Materials stable, even under fire exposure conditions, and not reactive with water. |
| 1 | Materials stable but can become unstable at elevated temperatures and pressures. |
| 2 | Materials undergoing violent chemical changes at elevated temperatures and pressures, reacting violently with water, or forming explosive mixtures with water. |
| 3 | Materials capable of detonation or explosive decomposition but requiring a strong initiating source must be heated under confinement before initiation, reacting explosively with water or detonating if severely shocked. |
| 4 | Materials readily capable of detonation or explosive decomposition at normal temperatures and pressures. |

**Table S.4.** Description of the pictograms used in Globally Harmonized System of Classification and Labelling of Chemicals.

| Pictogram | Hazard statements |
| --- | --- |
| 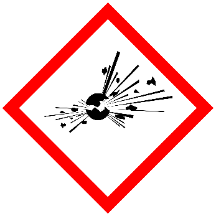  GHS01:  Explosive | H200: Unstable explosive  H201: Explosive; mass explosion hazard  H202: Explosive; severe projection hazard  H203: Explosive; fire, blast or projection hazard  H204: Fire or projection hazard  H209: Explosive  H210: Very sensitive  H211: Maybe sensitive  H240: Heating may cause an explosion  H241: Heating may cause a fire or explosion |
| 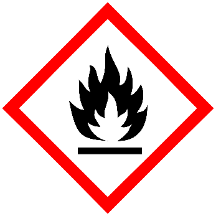  GHS02:  Flammable | H205: May mass explode in fire  H206: Fire, blast or projection hazard; increased risk of explosion if desensitizing agent is reduced  H207: Fire or projection hazard; increased risk of explosion if desensitizing agent is reduced  H208: Fire hazard; increased risk of explosion if desensitizing agent is reduced  H220: Extremely flammable gas  H221: Flammable gas  H222: Extremely flammable aerosol  H223: Flammable aerosol  H224: Extremely flammable liquid and vapor  H225: Highly flammable liquid and vapor  H226: Flammable liquid and vapor  H228: Flammable solid  H229: Pressurized container: may burst if heated  H230: May react explosively even in the absence of air  H231: May react explosively even in the absence of air at elevated pressure and/or temperature  H232: May ignite spontaneously if exposed to air  H241: Heating may cause a fire or explosion  H242: Heating may cause a fire  H250: Catches fire spontaneously if exposed to air  H251: Self-heating; may catch fire  H252: Self-heating in large quantities; may catch fire  H260: In contact with water releases flammable gases which may ignite spontaneously  H261: In contact with water releases flammable gas  H282: Extremely flammable chemical under pressure: may explode if heated  H283: Flammable chemical under pressure: may explode if heated |
| 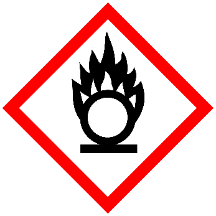  GHS03:  Oxidizing | H270: May cause or intensify fire; oxidizer  H271: May cause fire or explosion; strong oxidizer  H272: May intensify fire; oxidizer |
| 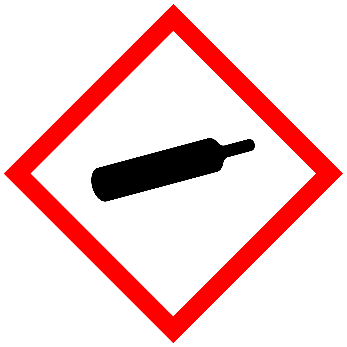  GHS04:  Compressed Gas | H280: Contains gas under pressure; may explode if heated  H281: Contains refrigerated gas; may cause cryogenic burns or injury  H282: Extremely flammable chemical under pressure: may explode if heated  H283: Flammable chemical under pressure: may explode if heated  H284: Chemical under pressure: may explode if heated |
| 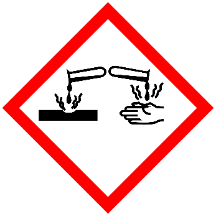  GHS05:  Corrosive | H290: May be corrosive to metals  H314: Causes severe skin burns and eye damage  H318: Causes serious eye damage |
| 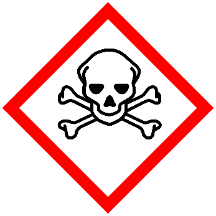  GHS06:  Toxic | H300: Fatal if swallowed  H301: Toxic if swallowed  H310: Fatal in contact with skin  H311: Toxic in contact with skin  H330: Fatal if inhaled  H331: Toxic if inhaled  H300+H310: Fatal if swallowed or in contact with skin  H300+H330: Fatal if swallowed or if inhaled  H310+H330: Fatal in contact with skin or if inhaled  H300+H310+H330: Fatal if swallowed, in contact with skin or if inhaled  H301+H311: Toxic if swallowed or in contact with skin  H301+H331: Toxic if swallowed or if inhaled  H311+H331: Toxic in contact with skin or if inhaled  H301+H311+H331: Toxic if swallowed, in contact with skin or if inhaled |
| 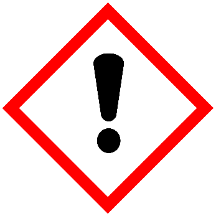  GHS07:  Harmful | H302: Harmful if swallowed  H312: Harmful in contact with skin  H315: Causes skin irritation  H317: May cause an allergic skin reaction  H319: Causes serious eye irritation  H332: Harmful if inhaled  H335: May cause respiratory irritation  H336: May cause drowsiness or dizziness  H420: Harms public health and the environment by destroying ozone in the upper atmosphere  H302+H312: Harmful if swallowed or in contact with skin  H302+H332: Harmful if swallowed or if inhaled  H312+H332: Harmful in contact with skin or if inhaled  H302+H312+H332: Harmful if swallowed, in contact with skin or if inhaled  H315+H320: Cause skin and eye irritation |
| 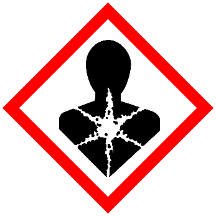  GHS08:  Health Hazard | H304: May be fatal if swallowed and enters airways  H334: May cause allergy or asthma symptoms or breathing difficulties if inhaled  H340: May cause genetic defects  H341: Suspected of causing genetic defects  H350: May cause cancer  H350i: May cause cancer by inhalation  H351: Suspected of causing cancer  H360: May damage fertility or the unborn child  H360F: May damage fertility  H360D: May damage the unborn child  H360FD: May damage fertility; May damage the unborn child  H360Fd: May damage fertility; Suspected of damaging the unborn child  H360Df: May damage the unborn child; Suspected of damaging fertility  H361: Suspected of damaging fertility or the unborn child  H361f: Suspected of damaging fertility  H361d: Suspected of damaging the unborn child  H361fd: Suspected of damaging fertility; Suspected of damaging the unborn child  H370: Causes damage to organs  H371: May cause damage to organs  H372: Causes damage to organs through prolonged or repeated exposure |
| 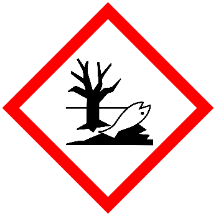  GHS09:  Environmental Hazard | H400: Very toxic to aquatic life  H410: Very toxic to aquatic life with long lasting effects  H411: Toxic to aquatic life with long lasting effects |





**Fig. S.1.** Chromatogram of the VOCs emitted from thermal degradation of HIPS during the procedure of mold preparation in investment casting.





**Fig. S.2.** Chromatogram of the VOCs emitted from thermal degradation of ABS during the procedure of mold preparation in investment casting.





**Fig. S.3.** Chromatogram of the VOCs emitted from thermal degradation of PLA during the procedure of mold preparation in investment casting.





**Fig. S.4.** Chromatogram of the VOCs emitted from thermal degradation of PP during procedure of mold preparation in investment casting.





**Fig. S.5.** Chromatogram of the VOCs emitted from thermal degradation of PA during procedure of mold preparation in investment casting.





**Fig. S.6.** Chromatogram of the VOCs emitted from thermal degradation of PMMA during the procedure of mold preparation in investment casting.
